# IoT-Based User-Driven Service Modeling Environment for a Smart Space Management System

**DOI:** 10.3390/s141122039

**Published:** 2014-11-20

**Authors:** Hoan-Suk Choi, Woo-Seop Rhee

**Affiliations:** Department of Multimedia Engineering, Hanbat National University, Daejeon 305-719, Korea; E-Mail: hkrock7904@gmail.com

**Keywords:** Internet of Things (IoT), user-driven service, service modeling, User Interface (UI), semantic, context aware

## Abstract

The existing Internet environment has been extended to the Internet of Things (IoT) as an emerging new paradigm. The IoT connects various physical entities. These entities have communication capability and deploy the observed information to various service areas such as building management, energy-saving systems, surveillance services, and smart homes. These services are designed and developed by professional service providers. Moreover, users' needs have become more complicated and personalized with the spread of user-participation services such as social media and blogging. Therefore, some active users want to create their own services to satisfy their needs, but the existing IoT service-creation environment is difficult for the non-technical user because it requires a programming capability to create a service. To solve this problem, we propose the IoT-based user-driven service modeling environment to provide an easy way to create IoT services. Also, the proposed environment deploys the defined service to another user. Through the personalization and customization of the defined service, the value and dissemination of the service is increased. This environment also provides the ontology-based context-information processing that produces and describes the context information for the IoT-based user-driven service.

## 1. Introduction

Recently, with the emergence of the IoT paradigm, the existing Internet environment has expanded with various things that connect with the Internet protocol and represent physical world entities. The IoT paradigm is applied to various services such as building management, energy-saving systems, surveillance services, smart homes, farm management, and so on. Users' needs have also become more complicated and personalized with the spread of user-participation services such as social media, blogging, *etc.* Some active users want to create their own Internet services. Therefore, Internet and web service providers, information technology (IT) device and appliance manufacturers, and third-party providers have opened their services and functions to the public in the form of open application programming interfaces (APIs). These enable everyone to generate newly converged services more easily [1]. The open API environment is effective only for service developers who have design ability and programming skills. Therefore, Internet service and product development are difficult for end users lacking programming skills, hardware development capabilities, and so on. Many user-friendly environments are actively progressing such as open-source physical computing platforms (e.g., Arduino, Raspberry pi and Intel Galileo) and open APIs. They provide an inexpensive and easy way to create devices through the simple integrated development environment (IDE) that runs on regular personal computers and allows users to write programs. Some people make their own electric products through this environment to satisfy their needs. In this way, the user-friendly environment is continuously evolving to satisfy the needs of the user.

Likewise, in the IoT service environment, users want to create their own specialized IoT services to fully satisfy the diverse goals in their daily lives. The IoT service consists of many service elements that are variables representing the situations of users such as related persons, available devices, required service behavior, statements of relevant device, *etc.* To provide an IoT service, we should describe service elements as a common and shareable form. Also, the service-creation environments should provide a rich expression to represent the daily lives of users through the composition of various service elements. The existing IoT service-creation environment enables users to create their own IoT services [2], but non-technical users find it hard to create services because the existing environment requires a programming ability (e.g., operation, code and script). Also, a non-technical user does not want to know the principles of service offering (e.g., service platform architecture, concept of ontology, criteria of context, required sensor type, service logic flow, *etc.*) [3]; so we need an easier way to create the IoT service.

To address these issues, we propose the IoT-based user-driven service modeling environment that consists of the user, the IoT service market, the IoT service platform and the service spaces. It is an environment aimed at lowering barriers for non-technical users to create their own IoT service without requiring any programming ability. In particular, it provides sequential service modeling to reduce error, confusion and context modification and to maximize rich expression. Also, through the IoT service market, we provide service registration, modification, personalization and brokering to spread the IoT service and its participation ecosystem.

The rest of paper is organized as follows: in Section 2, we discuss the related researches. In Section 3, we propose the IoT-based user-driven service modeling environment. It includes the base ontology for the IoT-based user-driven service, the ontology-based context-information processing process and the user-driven service modeling process. In Section 4, we show the implementation results of the proposed environment for a smart space management system. It shows the user interface (UI) progress and results according to the target scenario. In Section 5, we discuss the comparison results with the related researches. Finally, we present the conclusions of our work.

## 2. Related Works

The IoT connects things to the Internet and provides context information which is machine readable on the web [4]. Through this new paradigm, the data generated by things is used to provide various smart space services. We studied several projects and researches focused on the following: (1) how to connect things to create the valuable context information; (2) how to exchange the valuable information with other solutions for various smart space services; (3) how to create an IoT service in an easy way.

The work by Guinard *et al.* [5] proposed a realization of the Web of Things (WoT). It provided representational state transfer (RESTful) APIs to integrate the services offered by devices and objects in the real world, such as wireless sensor networks, embedded devices and household appliances, with any other web content. It represented devices, objects and web services as resources of the web. Also, Guinard *et al.* showed two concrete implementations: on the Sun SPOT platform and on the Ploggs wireless energy monitors. However, they did not consider semantic heterogeneity to provide interoperability with other solutions and to allow non-technical users to create services in an easy way. Also, the service description needs to be modified according to each environment.

The UbiSOA by Avilés-López *et al.* [6] presented a framework and user-interaction model for IoT applications based on the technologies of the modern web. It provided a service description and discovery, event notification, execution language, and engine and service mash-up editor. Also, Avilés-López *et al.* implemented a working prototype and test applications for a variety of scenarios. The mash-up environment requires execution recipes and the application manifests as extended personal hypertext preprocessor (PHP) code to execute the service, but a non-technical user does not have the programming ability and does not understand the concept of this framework. Therefore, a more intuitive and easy service mash-up editor is required.

The work by Han *et al.* [7] presented a building automation system (BAS) adopting the service-oriented architecture (SOA) paradigm with devices implemented by Devices Profile for Web Services (DPWS) [8]. Also, it proposed a description of the context, composition plan and predefined policy rules. It composed a relevant context-aware service according to the context-matching algorithm through the six-phased composition process. They observed the context by ontology and defined the service as one function of the actuator. Han *et al.* provided an implementation prototype—the SamBAS, based on DPWSim—and showed the experimental result of the service execution time with the service cache. The service cache stores context-matching results and provides them to the service binder and service executor in the composition process. It improves the service execution speed, but if the context and service requirements are complicated, the creation of the composition plan will be difficult. Also, the service cache hit ratio is low, and non-technical users find it difficult to create services.

The work by Quyet [9] proposed the hybrid semantic energy-saving service for cloud-based smart homes. It included three approaches. In the first approach, it visualized the energy consumption of the device, home and system context in order to support consumers in making decisions to manually control the devices for energy saving. The second approach considers energy-saving rules. It is created from device, home, and system energy-saving policies, so the smart home system provides automatic control with predefined rules. The last approach was the ability to self-adapt and personalize by checking the user pattern. It found possible periodic and sequential patterns of user behaviors and automatically converted them to the automatic rules to adopt this pattern into the service rule. However, tracking and recording the activity of the user increases processing costs.

ClickScript [10] allowed the user to visually create a Web 2.0 mash-up by connecting building blocks of resources (e.g., web pages, string, *etc.*) and operations (e.g., greater than, if/then, *etc.*). The user can create a simple mash-up service through drag and drop, but if the user does not know the meaning of operations and building blocks, it is difficult to create a mash-up service. Also, the user should know the relevant object type and criteria of the service condition.

JIGSAW [11] is a tablet-based editor that provides the jigsaw pieces to support service dynamic recombination for end-users. It expressed the various functional modules as the jigsaw puzzle pieces. The use of jigsaw pieces allows users to develop functions through a process of left-to-right assembly. It argued the following: (1) the users can readily understand components as jigsaw pieces; (2) the users understand the concepts involved in building assemblies of devices; (3) the users can make assemblies of components to meet their local needs; and (4) the users can suggest additional devices that can fit into the overall framework and metaphor. However, there is the possibility of misunderstanding the metaphor according to the user levels and characteristics. Also, it cannot define a service which requires arithmetic operations, because it does not support the relational and arithmetic operations.

## 3. IoT-Based User-Driven Service Modeling Environment

### 3.1. IoT-Based User-Driven Service

**Figure 1.**
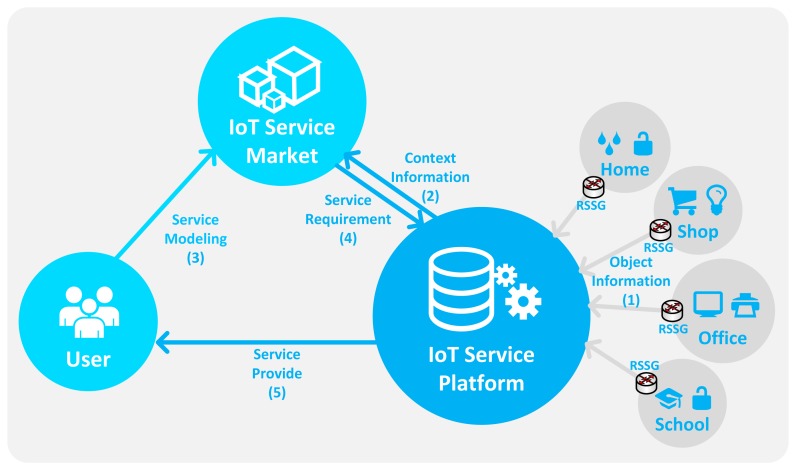
Overview of the IoT-based user-driven service environment.

We define the IoT service as a service provided by context information generated by a sensor (or device). This sensor (or device) is connected, managed and controlled by IoT technology. Because of the user-centric and participatory paradigm, such as App Store, Open Web and social network services (SNSs), some active users want to apply their needs to the service. In an IoT-based smart space management service, such as a BAS or energy-saving system, the user needs are varied and complicated because there are many situations and things to consider in order to process the data around the user that is required to provide context awareness. To satisfy the various needs of the user, the user-centric and participatory paradigm will be applied to the IoT-based smart space management service. Therefore, we propose the IoT-based user-driven service. It is the IoT-based smart space management service that was defined by one user and deployed to another user to increase its value through the service personalization and customization. It should be able to express a variety of conditions of the user because the user can be located in various places and faced with many situations. Also, it provides a user-friendly environment to support non-technical users. The proposed service environment is shown in Figure 1.

It consists of the user, the IoT service market, the IoT service platform and the service spaces. In the IoT service, the user has a relation with various spaces (such as school, office, shop, home, *etc.*). These spaces include multiple places (such as classrooms, offices, restrooms, laboratories and corridors) that contain various objects (such as lamps, heaters, roll screens, projectors, curtains, air conditioners and ventilators). To provide context awareness, we observe the phenomenon around the user through the relevant object. But these objects communicate with different protocols and networks, so the data generated by each device has heterogeneity. To solve this problem, we propose the RESTful smart space gateway (RSSG) that provides multiple communication protocols and the object data translation to represent object data as the web standards. In the proposed environment, the RSSG is placed in every space, where it aggregates, manages and sends the object information (1) to the IoT service platform as an abstracted common format. The IoT service platform manages and processes the aggregated information for context awareness through the ontology. Also, the removal of the semantic heterogeneity of aggregated information is required to apply it correctly in a variety of service domains. In Section 3.2, we propose the base ontology to describe the context information (2) required to describe the service. We also propose the procedures for the ontology-based context-information processing in Section 3.3. It describes the whole procedure of processing—from aggregating object data to context information, and classification of the context information.

We also propose the IoT service market to provide the user-driven service modeling process in Section 3.4. It describes service elements and how to model the IoT service. The IoT service market provides a web-based service modeling UI to the user for service modeling (3), and the created IoT service is stored and registered in the service repository of the IoT service market to be deployed and modified by another authorized user. Also, the IoT service market abstracts the service requirement (4) that is service modeling result to deliver to the IoT service platform. The IoT platform applies this requirement to the base ontology. It processes the context information and service requirement to provide the IoT service to the user (5). If the service condition is satisfied, the IoT platform controls the relevant actuators according to the service requirement.

Through the proposed method, the proposed environment provides the following features:

- Predefined context: we provide a predefined context to the user to support a simple and easy method of service condition definition.
- Context modification and personalization: we provide context modification and personalization to support easy context reconfiguration to satisfy the criteria of personal needs.
- Context mash-up: we can define a more complicated phenomenon and substantial situation model through the context mash-up regarding existing context that was predefined, modified and personalized by the proposed environment.
- Reusable service element: we can create a new service element through the combination of the existing service element that was defined by you and another user.
- Sequential service modeling and no requirement for programming ability: the sequential service modeling reduces the mistakes of the user with regard to the service modeling. It only shows the user the possible elements in each phase. We do not require any programming ability to support a non-technical user.

These features are the main characteristics of our environment. So, focusing on these features, we analyze the related works in Section 5.

### 3.2. Base Ontology for IoT-Based User-Driven Service

**Figure 2.**
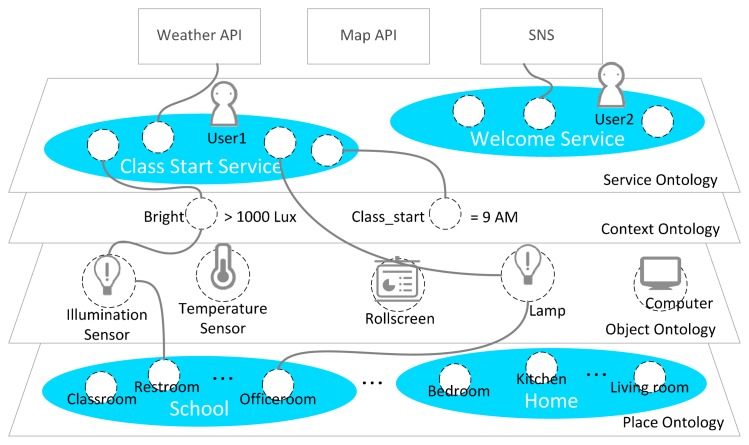
Base-ontology overview.

Ontology is defined as an explicit and formal specification of conceptualization [12]. We need ontology to define sensor data in order to provide context-aware IoT services and to describe the virtual world that represents the service domain. Figure 2 shows the proposed base ontology overview. It consists of four layers as follows:

- Place ontology: This ontology describes service-domain characteristics such as home, school, shopping mall and office. For example, in the home service domain, it describes numerous places such as the kitchen, living room, bedroom, *etc.* Also, the place ontology can expand with a hierarchical structure by linking to another place ontology.
- Object ontology: This ontology describes sensors and actuators to represent real-world entities such as lamps, computers, roll screens and temperature sensors. The sensors link with the observation value by the *hasValue* property to describe the measurement value. The actuators link with the status value by the *hasStatus* property to describe the available function and status information. Also, all objects have a location linked with the place ontology.
- Context ontology: The service situation describes a certain phenomenon. It is defined by a link with relevant sensors that are able to generate a value to determine a certain phenomenon. Therefore, the context consists of the observation value and rule that are the criteria of the context. Also, context location is recognized by the location of the relevant sensor.
- Service ontology: It is comprised by the context and object ontology. It describes the service condition and service behavior.

### 3.3. Procedures for the Ontology-Based Context-Information Processing

**Figure 3.**
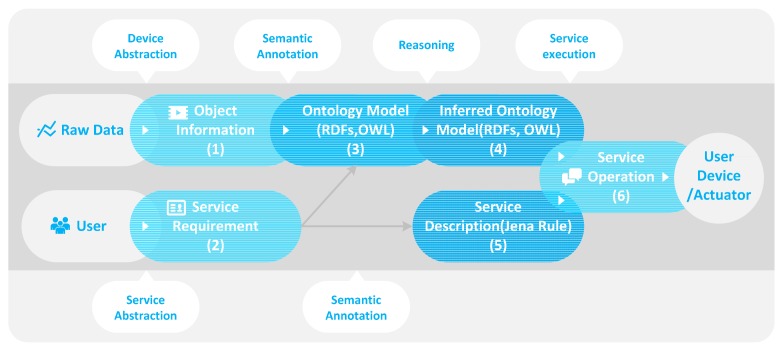
Procedures for the ontology-based context-information processing.

Figure 3 shows the procedures for the ontology-based context-information processing. It is a procedure for producing and describing the context information used in the IoT service as a service element. Also, it shows how to determine the service condition and execution. This procedure consists of the device abstraction, the semantic annotation, the reasoning and the service execution. Through the device abstraction, the raw data that comes from the object is abstracted as a common format to remove heterogeneity and to reuse other services.

The abstracted data is represented to the object information as shown in Figure 3(1). Also, when the user creates a new service through the web-based service modeling UI, the IoT service market abstracts the service modeling result that is represented to the service requirement as shown in Figure 3(2). The object information (1) and the service requirement (2) maps the ontology model (3) to describe the service element and IoT service through the semantic annotation. Based on this ontology model, the context information required by the service condition is processed. It includes some rules and the context information that is represented to the service description as shown in Figure 3(5). The reasoner makes the inferred ontology model (4) that is a classified result based on the ontology model (3). By this inferred ontology model, we can determine the service condition and create the service operation (6) of the service behavior according to the service description (5).

### 3.4. User-Driven Service Modeling Process

To provide a context-aware IoT service, the definition of context and service-offering conditions is very important because it is the basis for the IoT service modeling. In particular, the service-offering conditions must be defined exactly to determine the service execution [3]. The existing service modeling environment provides mash-up editors to the end-user to create their own services. It can illustrate the integration of things (sensor or actuator) with simple operation or code (e.g., AND, OR, greater than, if/then, loops, *etc.*). But a non-technical user does not know how to define service with simple operation or code. Also, they do not want to know the principles of service offering (e.g., service platform architecture, concept of ontology, criteria of context, required sensor type, *etc.*).

Therefore, we propose the user-driven service modeling process that provides an easier service modeling environment for a non-technical user. Using this process, the user decides on only three main elements: service domain, actuator's function and service condition for modeling of the IoT service.

- Service domain: It represents the IoT service domain that includes the space and object information. The user can create or modify the service domain. Also, the user defines the actuator's function and service condition through the space and object information that are described by the service domain.
- Actuator's function: It is a result of the service the user wants. It defines the selected actuator's behavior.
- Service condition: It consists of a few pieces of context information that are predefined by the platform. The user can create or modify the context information when the service condition is defined. It works as the criteria of the service execution.

**Figure 4.**
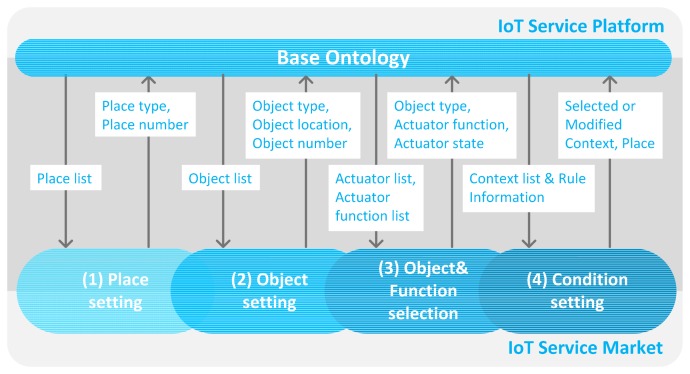
User-driven service modeling process.

Figure 4 illustrates the user-driven service modeling process including data acquired by query from the base ontology. The arrow in Figure 4 associated with the IoT service market in the base ontology is a query result which includes necessary information of each phase. Also, the arrow in the opposite direction is the result of each modeling process. It consists of four phases. First of all, we need to define the service domain to represent the variety of spaces such as the type and number of places. For example, schools have many classrooms, offices, laboratories, restrooms, *etc.* It can be annotated by the user because it cannot be classified automatically by some sensor or device. Also, the objects require location information to elicit the place condition. We annotate location information to the discovered object. Therefore, we define the place type of each space and select the object, and then the location information is annotated to the relevant object dynamically. To define the service domain, two phases are performed: (1) the place setting, and (2) the object setting. After this phase, the service modeling is performed with two phases: (3) the object and function selection, and (4) the condition setting. In the object and function selection phase, the user selects the actuator and its function to define the service behavior. Also, in the condition setting phase, the user selects and/or creates and/or modifies the context to define the service condition. The proposed process provides the predefined context and the reusable service elements by sequential process to define the IoT service by a non-technical user. To do this easily, we provide the web-based service modeling UI.

## 4. Implementation Result of Proposed Environment for Smart Space Management System

### 4.1. Target Service Scenario

We introduce the target service scenario in the school domain to describe the proposed environment. We assume that the school includes some spaces such as two classrooms, one office room, two restrooms, one laboratory and one corridor. Each space has various objects such as lamps, heaters, roll screens, projectors, curtains, air conditioners and ventilators. It also has various sensors to acquire context information such as the classroom being hot and dark. We define the target service scenario as follows:

1. At 9 a.m., the user wants to prepare the devices that are necessary for class. For example, the projector and computer are activated and the roll screen is turned on. In addition, if the class is too bright to see the projection image, the curtains are closed.
2. If the classroom is too hot at five minutes before class time, the user wants to turn on the air conditioner.

This scenario includes multiple context information about time, temperature, and illumination to define the service condition. Also, it contains some actuator functions to define the service result. To manage this information, we need to aggregate and control the object that is able to classify by the sensor and actuator.

### 4.2. Implementation Environment

The user has relationships with a variety of spaces and has many devices that communicate with different protocols and networks. The data generated by each device has heterogeneity such as system, syntax, structure, and semantics. It means that sharing and reuse of sensor data is difficult within such a heterogeneous device communication environment [13].

To solve this problem, we propose the object data (abstracted data) format to remove heterogeneity of syntax and structure. Table 1 is the proposed object data format. The object is classified into the sensor and actuator. Also, the object data is addressed by the uniform resource identifier (URI) to be controlled, managed and used by the RESTful operation. The sensor data include URI, type, unit, value, accuracy, location and ownership for measurement. Actuator data include URI, name, status, function list, location and ownership to control. The object data are deployed based on JSON [14]. However, JSON does not provide any means to describe semantics. When object data is received, we understand that it has a certain type of value such as a temperature value of 28 °C. However, the value can be the body temperature of a person or room temperature, so we need the semantic annotation to process the object data to the context information. We describe semantics on the object data through the proposed ontology-based context-information processing as shown in Figure 3.

**Figure 5.**
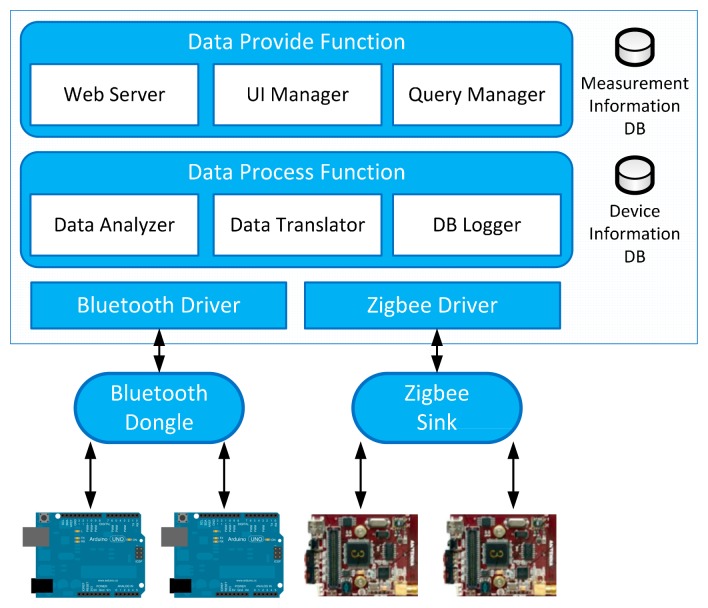
Implementation architecture of the RESTful smart space gateway (RSSG).

Figure 5 is the implementation architecture of the RSSG. It is deployed at each service domain to aggregate object data. It is implemented by Hypertext Markup Language (HTML5), Cascading Style Sheets (CSS3), PHP5.5, JavaScript, Java Enterprise Edition (EE), RxTx library [15], Slim framework [16], MySQL 5.1 and Apache web server. The RSSG prototype is developed in the Java to provide data aggregation, object management, and data translation functions. To support various types of interface, we use multiple interface drivers (Bluetooth and Zigbee) to communicate with various types of wireless communication protocols. We programmed all sensor nodes to detect every two seconds and send the sensor data to the RSSG. When the RSSG receives the sensor data, the data process functions analyze the sensor data through the acquired device information by device information Database (DB). The device information includes the device type and the required data type of the relevant device to manage the connected devices. The data translator encodes sensor data according to the object data format as shown in Table 1. If the incoming sensor data is the same as the sensor data that arrived previously, we do not need to translate and update it. The DB logger checks the variation of the value to minimize the processing cost. The translated data (abstracted data) is stored in the measurement information DB. When the RSSG receives the sensor data request through the specific URI, the web server of the RSSG processes the sensor data request based on the RESTful method, and the query manager acquires the requested information from the measurement information DB.

We aggregate sensor data through two types of sensor node: one is the Arduino UNO 3.0 with a Bluetooth shield, and the other is the Zigbee sensor node. They connect temperature, humidity and motion detection sensors. Arduino has different versions of the original model with various capabilities that have been developed (UNO, Mega, Leonardo, Mini, Due, Yún, *etc.*) [17]. We selected the newest one: the Arduino UNO. Also, we added the Bluetooth shield to the Arduino UNO. The other node is the Zigbee sensor node, and it was selected because it is one of the most commonly used wireless communication protocols. This node embeds temperature, illumination, humidity and infrared sensors [18].

For convenience, we implement the IoT service platform and IoT service market on one computer, but they are separated, conceptually, by a process unit, as shown in Figure 1, and they communicate with each other by REST operation and socket communication. We develop the prototype in Java EE and use the following technologies:

We use Apache Tomcat 6.0 to support the RESTful interface, the Jena framework 2.6.4 [19] for processing semantic data, SPARQL API to query semantic data, Pellet [20] to support semantic reasoning, Jena rule for describing service rules, Jackson API 2.2.3 [21] to convert the Java object to JSON and MySQL 5.1 for storing and loading ontology.

### 4.3. Ontology-Based Context-Information Processing Result

In the ontology-based context-information processing, the sensor data is aggregated and abstracted as the object-data format through the RSSG as described in Table 1. Also, the object data converts to context information to provide the IoT service. This processing consists of four phases: device abstraction, semantic annotation, reasoning and service execution. We show the processing results according to the smart school service scenario as described in Section 4.1.

#### 4.3.1. Device and Service Abstraction

**Figure 6.**
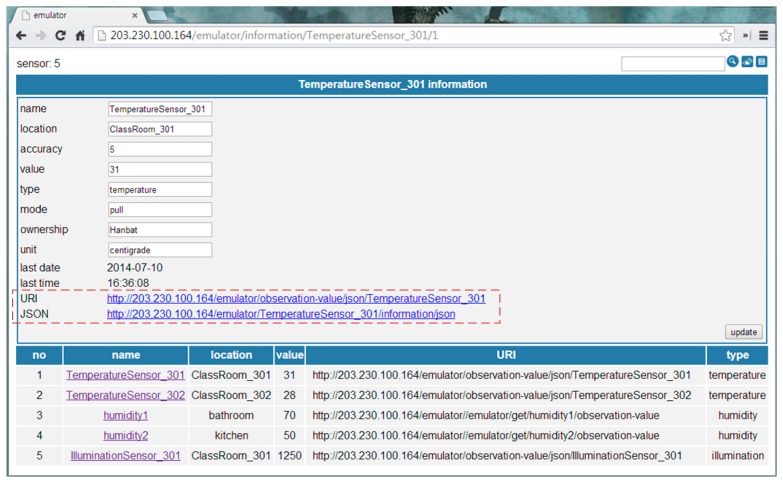
Monitoring UI of the RESTful smart space gateway.

When a device is on, it sends measured data to the RSSG. Then, the RSSG detects the object type using the source address and translates to the object-data format, according to the device information, dynamically. Figure 6 is the object-data monitoring UI of the RSSG. It shows detailed information of the abstracted object data (*i.e.*, temperatureSensor_301) at the upper part of the UI. It includes location, accuracy, value, type, mode, ownership, unit and update time. Also, the RSSG provides two types of URI that are shown in the part of the dotted rectangular box of Figure 6. The first type of URI requests all detailed information of the abstracted object. It is used to initialize the new object. The second type of URI requests only the observation value to minimize the cost of the value update. The bottom of this UI shows the available object list and its information as a table. The service abstraction is a process that describes the service requirement as a common format. Also, it is a result of the user-driven service modeling process by the web-based service modeling UI.

**Figure 7.**
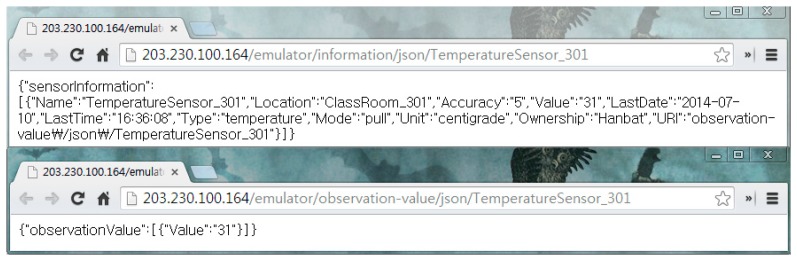
Abstracted object data.

Figure 7 shows the abstracted device data as the JSON format. It is an example of Figure 3(1) that is generated by the temperature sensor.

It includes sensor information (*i.e.*, name, location, accuracy, value, time, type, ownership and unit) as shown in the upper part of Figure 7. The bottom of Figure 7 shows the observation value to minimize the data size. Therefore, we know that *ClassRoom_301*'s temperature is 31 °C.

#### 4.3.2. Semantic Annotation

**Figure 8.**
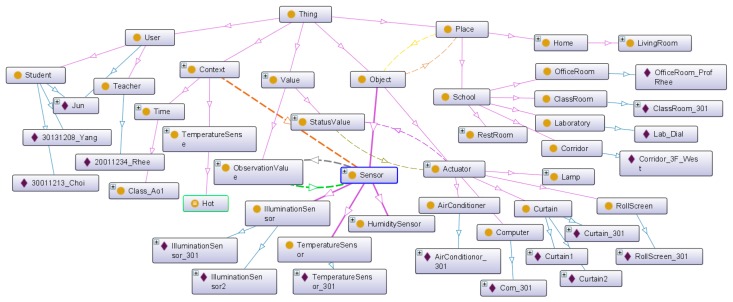
Base Ontology.

When the IoT service platform receives the abstracted object data, we need the semantic annotation to describe the semantics. Figure 8 shows the proposed base ontology by the OntoGraf [22] tool that is the ontology graphic view plugged in Protégé. The proposed ontology consists of place, object, context user and value class. As shown in Figure 8, we define some available objects in the service domain. The *Actuator* class has various types of subclass to classify actuator types such as heaters, computers, roll screens, air conditioners and curtains. Each subclass of the *Object* class has the real-world entity as the instance according to the target scenario. Also, the *Place* class represents the target space that is the service domain. It contains the ClassRoom, Corridor, Laboratory, Restroom and OfficeRoom, and it has the real-world place as the instance. The *Sensor* class has a *hasValue* property that is linked with the *ObservationValue* class. The value of the sensor data updates to the literal that was linked with the relevant sensor instance (e.g., *TemperatureSensor1*). Then, we know that the temperature of a particular place (e.g., *ClassRoom_301*) is 31 °C. To define the context, we linked the *hasSensor* property with the *Context* class and the *Sensor* class. Also, we know the location of the context through the location of the relevant sensor. As a result, the abstracted device data is mapped to the context information such as “The *ClassRoom_301* is hot”.

**Figure 9.**
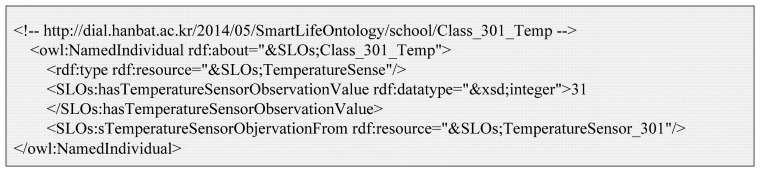
OWL description of *Class_301_Temp* context.

Figure 9 is the OWL description of the example context (*Class_301_Temp*). It is a member of the *TemperatureSense* class that is a subclass of the *Context* class. It is part of ontology model as shown in Figure 3(3). The *Class_301_Temp* instance is linked with *TemperatureSensor_301* by the *'sTemperatureSensorObjervationFrom* property. Also, it has the current temperature value by the *hasTemperatureSensorObservationValue* property.

#### 4.3.3. Reasoning

**Figure 10.**
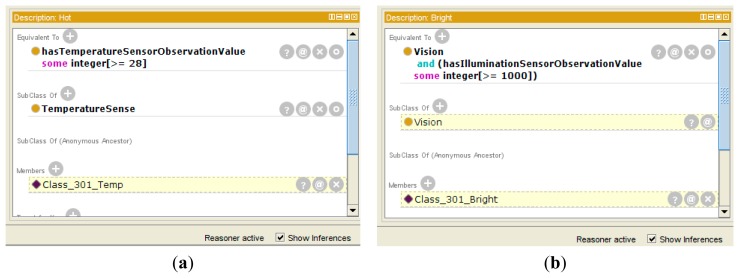
Inferred ontology model. (**a**) *Class_301_Temp* instance; (**b**) *Class_301_Bright* instance.

A class in OWL is a classification of individuals into groups that share common characteristics. If an individual is a member of a class, it tells a machine that it falls under the semantic classification given by the OWL class. This provides the meaning of the data that allows the reasoning engine to draw an inferred model from the base ontology model. Figure 10 shows the inferred ontology model of the *Hot* class and the *Bright* class that are examples of Figure 3(4). The inferred ontology model is a result of the reasoning by the Pellet reasoner [20] that is integrated with the Jena library for inference functionalities. Each class includes some restriction of defining the characteristics or criteria of a certain phenomenon. For example, we define the *Hot* context as having a temperature value larger than 28, and the *Bright* context as having an illumination value larger than 1000. To personalize each context, we define the class and contain the relevant context instance that is linked with the observation value. If the *Class_301_Temp* instance has a temperature value of 31 through the *TemperatureSensor_301*, it is classified as a member of the *Hot* class because the observation value is larger than 28. Also, the *Class_301_Bright* instance is classified as a member of the *Bright* class according to its criteria.

#### 4.3.4. Service Execution

**Figure 11.**
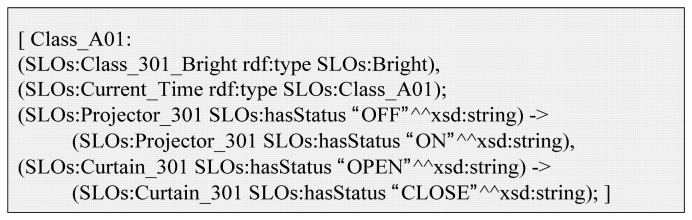
Service rule description (Jena rule).

Finally, service is executed by the IoT service platform based on the Jena framework [19]. Figure 11 shows the service rule description that is an example of Figure 3(5). We define a SPARQL query that is able to acquire a satisfied member of the service condition, and an execution query by the thread of the Java prototype.

When the *Class_301_Bright* instance is classified as a member of the *Bright* class, and the *Current_Time* instance is classified as a member of *Class_A01*, the service condition is satisfied. When the service execution thread acquires the query result, the thread compares it with the service rule to determine if the service conditions are satisfied. If the service execution condition is satisfied, the relevant actuators have some status. For example, *Projector_301* has status “ON”.

If the status of the actuator has changed, we update the status using the REST PUT operation. It is a simple way of updating a value. Figure 3(6) is the status update message according to service execution result. It is updated by the URI of the target actuator (*i.e*., http://hostaddr/put/actuator/projectoer_301/status/on).

### 4.4. User-Driven Service Modeling Process with the Web-Based Service Modeling UI

We provide the web-based service modeling UI to create the user-driven service without any programming ability or study of the criteria of the phenomena required as a service condition. This UI is implemented with HTML5, CSS3 and JavaScript. Data exchange with the IoT service platform is performed by HTTP and JSON. We arrange the process state bar at the top of the screen to recognize the current phase. The user can create a target service through the sequential progress that consists of four phases (Place setting, Object setting, Object and function selection and Condition setting) according to the user-driven service modeling process as shown in Figure 4. We show the UI progress, query result and description of ontology according to the target scenario (1) as described in Section 4.1.

#### 4.4.1. Place Setting

**Figure 12.**
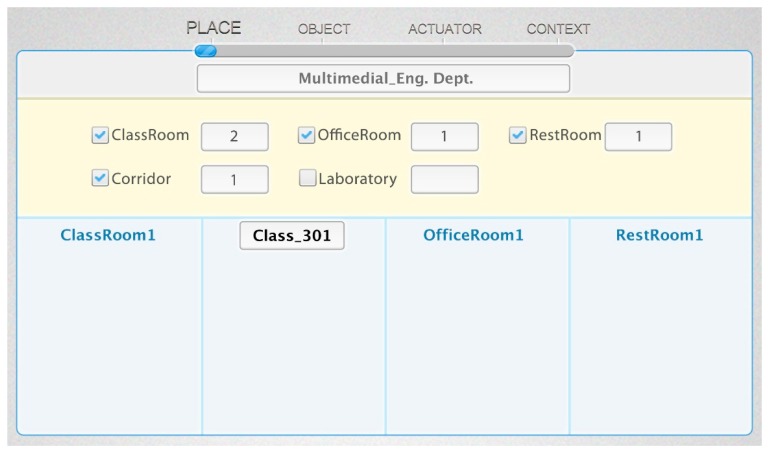
Web-based service modeling UI (Phase 1: place setting).

In this phase, we define the service domain, such as type of place (e.g., classrooms, offices, restrooms, laboratories, corridors, *etc.*), and the number of each type. We define characteristics of each space by the base ontology. First, the user enters the name of the service domain to create a new service environment. When the user selects the type of place and enters the number of places, the real-world place will be added to the ontology as an instance. The upper part of Figure 12 shows the available place types, and the lower part shows the created place by the user. According to the target scenario, we create two classrooms, one office, one restroom and one corridor. Also, the user is able to modify the name of each created place to personalize them—such as *Class_301* as shown at the bottom of Figure 12.

**Figure 13.**
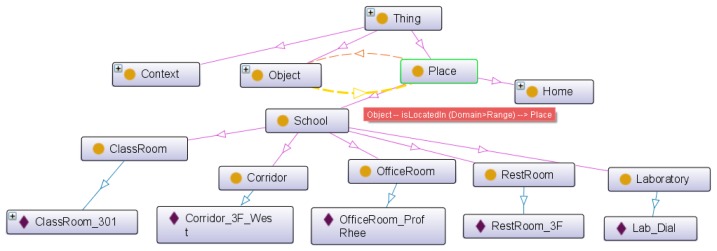
Base ontology (Place instance).

Figure 13 shows the proposed base ontology by OntoGraf. It shows the result of place setting that includes an instance of each place class.

**Figure 14.**
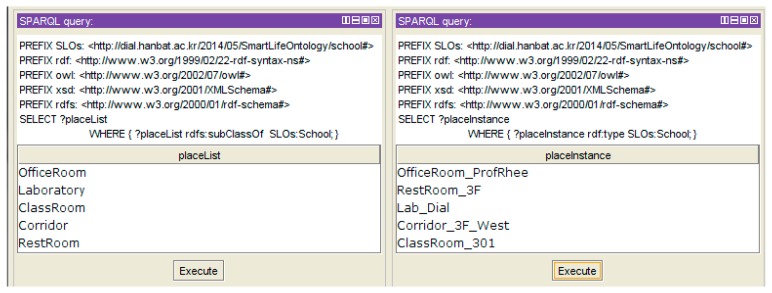
Examples of place query result

Figure 14 shows the SPARQL query result. The web-based service modeling UI is able to obtain the place type and instance through the SPARQL query. The “SLOs” is the name space of our ontology.

#### 4.4.2. Object Setting

**Figure 15.**
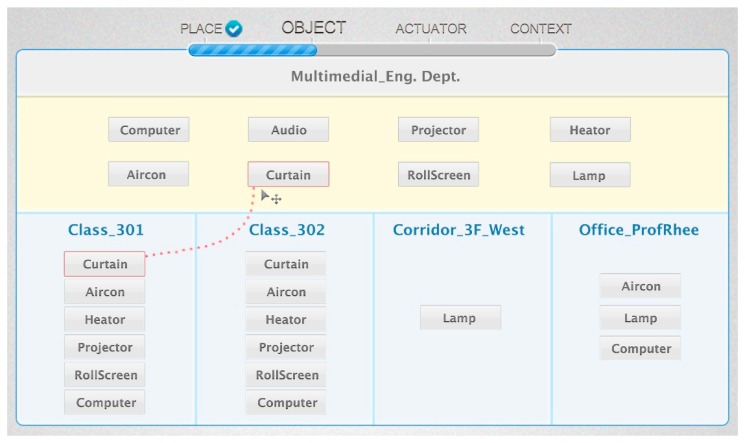
Web-based service modeling UI (Phase 2: object setting).

When the IoT service platform receives the abstracted object data, the object data is added to the base ontology as an instance. The user places the real-world physical objects according to the list of available objects of each place deployed by the IoT service platform. The upper part of Figure 15 shows the available actuators, and the lower part shows the result of the object setting. The user can place objects through drag and drop.

**Figure 16.**
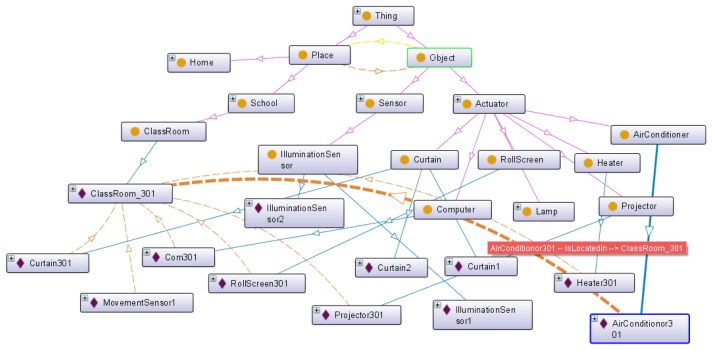
Base ontology (Object instance).

The object-setting result is described as a JSON message by the IoT service market. The message is transferred to the IoT service platform to create instances on the base ontology. Figure 16 shows the result of the object-setting phase with the base ontology statement. The *ClassRoom_301* instance is linked with the relevant object instance by the *hasActuator* property. Also, all object instances are linked by the *isLocatedIn* property to define the location information, so we know that the location of the *Curtain_301* instance is *ClassRoom_301*. After the object-setting phase, the place and object-setting phases are no longer required to create a new service with the same environment. Also, if the environment is changed, the user can modify or create the new environment.

**Figure 17.**
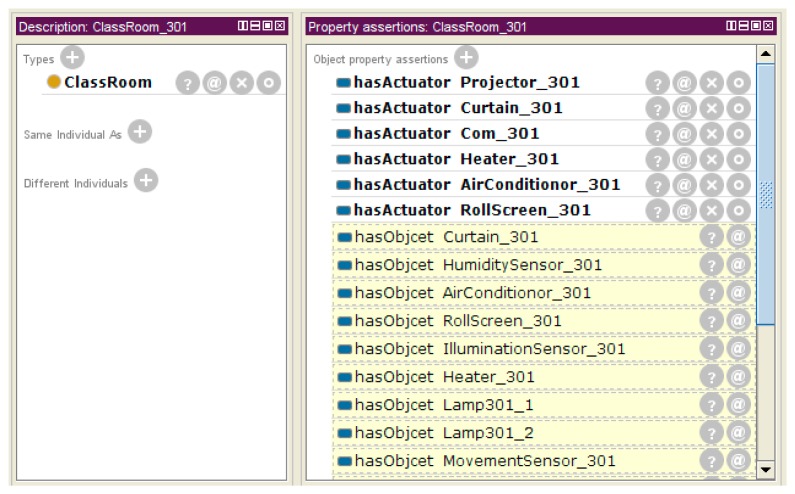
Object instance example in *ClassRoom_301*.

Figure 17 shows the description of the *ClassRoom_301* instance by the Protégé description view. The *Place* instance has some objects as a result of the object-setting phase. *ClassRoom_301* has actuators (e.g., *Projector_301*, *Curtain_301*, *Com_301*, *Heater_301*, *AirConditionor_301* and *Rollscreen_301*) and objects (e.g., *HumiditySensor_301*, *IlluminationSensor_301* and *MovementSensor_301*) to represent the service domain.

**Figure 18.**
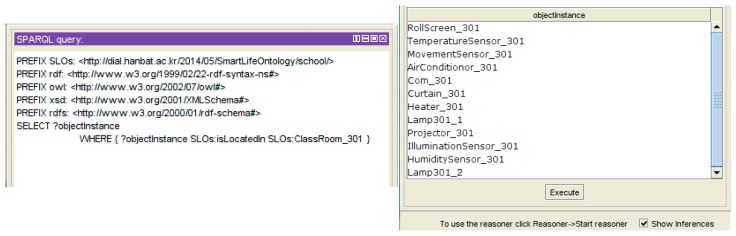
Example of object query result.

When the IoT service platform acquires the object information to process the service condition or service execution, the web-based service modeling UI queries the object list related with the target service place as shown in Figure 18. It shows the connected object list. The UI shows the existing object information through this SPARQL query.

#### 4.4.3. Object and Function Selection

**Figure 19.**
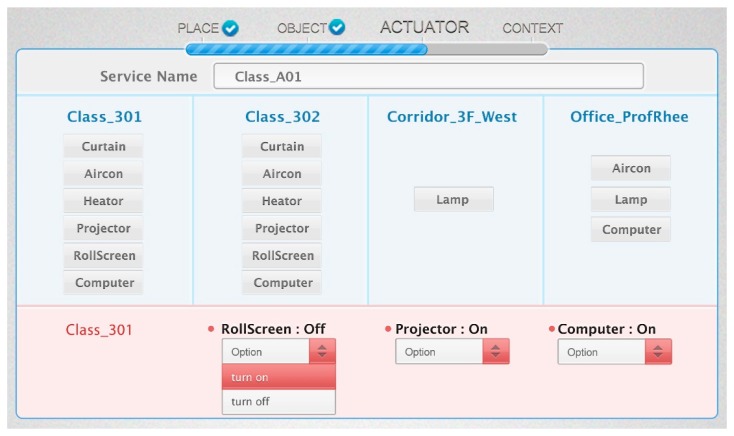
Web-based service modeling UI (Phase 3: object and function selection).

In this phase, we define the service behavior desired by the user. First, the user enters the service name to create a new service. The user selects the actuator's function for the required behavior by the user (e.g., open the window, turn on the projector). Figure 19 shows the UI to select the object and function. The upper part of the screen shows the available actuators in their location. When the user selects the location, the UI shows the available objects through the object query as shown in Figure 18. The lower part of the screen shows the function list of the selected actuator. The user can create a service behavior through the selected function. We select “turn on” function of *RollScreen* according to the target scenario. The user can execute many functions in the same service condition by selecting multiple actuators and their functions. Selecting many functions represents the “AND” operation between the selected actuator's functions.

**Figure 20.**
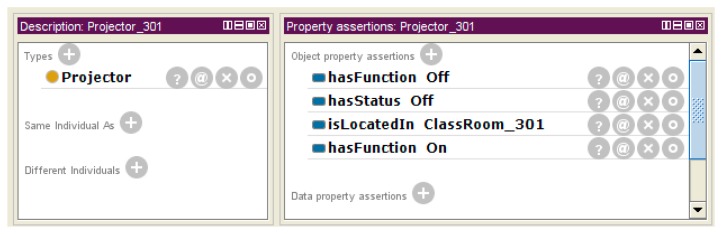
Actuator instance example, *Projector_301*.

Figure 20 shows the description of the *Projector_301* instance by the Protégé description view. It includes the “On” and “Off” functions. We know the available function list of each actuator by using the *hasFunction* property, and we also know the status information of the object by using the *hasStatus* property.

#### 4.4.4. Condition Setting

**Figure 21.**
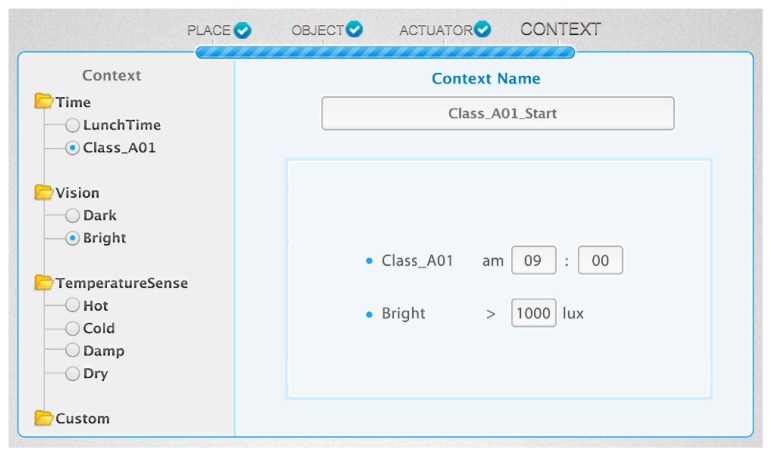
Web-based service modeling UI (Phase 4: condition setting).

In this phase, the user selects a context to define the service condition. Some pre-defined contexts are provided by the IoT service platform as the categorized form shown in the left side of Figure 21. When the user selects the context, the service modeling UI shows the detailed information of the context such as the criteria of each context. For example, the time of *Class_A01* is defined as 9 a.m. and the *Bright* category of the class is defined as larger than 1000 lux. Then, the user does not need to know if a particular context has any criteria of observation value and required sensor type. The user can also create a new context using the existing context. For example, if the user wants multiple conditions, such as class time and brightness, then the user just selects multiple contexts such as those in Figure 21. As a result, the customized context is added to the custom category of context. The user can also create a new context category and add a created context to extend the context. This can also be applied to other services when the user wants the same situation. We provide pre-defined contexts in a categorized form such as time, vision, temperature sense and custom that has disjointed characteristics with each other. Therefore, the user does not make a mistake when the new context is created.

**Figure 22.**
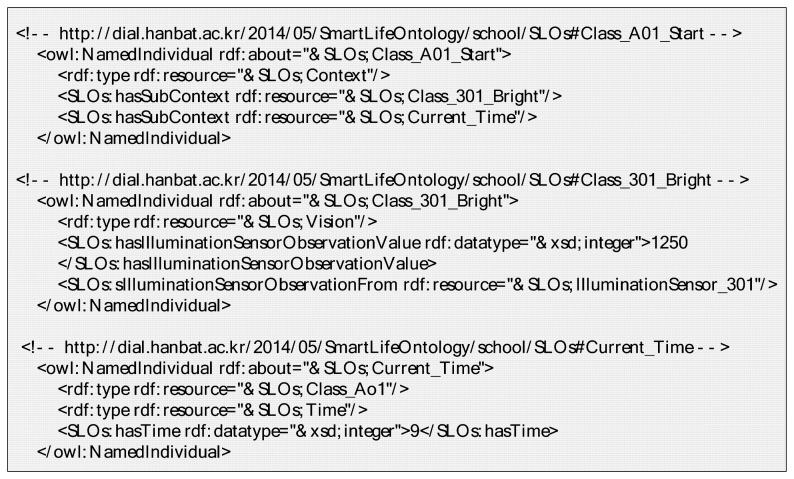
OWL description of *Class_A01_Start* with its sub-contexts.

Figure 22 is the OWL description of the *Class_A01_Start* instance created through the condition-setting phase as shown in Figure 21. It is a part of the ontology model shown in Figure 3(3). The *Class_A01_Start* instance has a sub-context of *Current_Time* and a *Class_301_Bright* instance. The *Class_301_Bright* instance is linked with the *IlluminationSensor_301* instance by the *hasIlluminationSensorObservationFrom* property. Therefore, it has a current illumination value by the *getObservationValueFrom* property to determine the context of the relevant place. Also, it is easy to reuse and personalize because the service condition is defined by the customized context, which includes various sub-contexts using the hierarchical structure.

**Figure 23.**
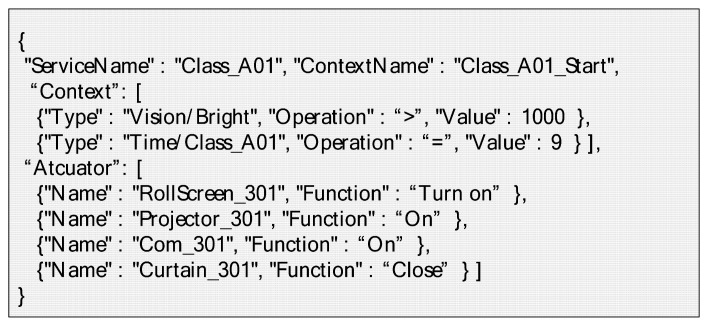
Example of abstracted service requirement.

When the user-driven service modeling process is finished, the requirement of service describes the abstracted format by JSON. The abstracted service requirement represents to the service condition and behavior that consists of the selected context, context criteria and the name of the actuator and function as shown in Figure 23. It is stored in the service repository of the IoT service market. Also, it can be customized and reused by another user.

## 5. Comparison Results

In this chapter, we provide analysis results of the comparison features between the proposed service modeling environment and the related studies as shown in Table 2. The comparison features are derived by the related work analysis and motivation of our proposed environment. It consists of six features (predefined context, context modification and personalization, context mash-up, reusable service element, sequential service modeling and programming ability not required). In this paper, we proposed three main ideas: the base ontology, the ontology-based context-information processing and the user-driven service modeling process with the web-based service modeling UI. Comparison features 1–4 are supported by the base ontology. The ontology-based context-information processing provides comparison features 4 and 6, and the user-driven service modeling process with the web-based service modeling UI provides all of the comparison features.

### 5.1. Predefined Context

The user is always faced with many circumstances, and these circumstances require very diverse elements to define them with rich expression. Also, the user does not know how to determine some circumstances such as “The living room is hot” and what kind of information is required. A simple circumstance makes it easy to elicit elements, but if the circumstance is uncommon or complicated, it needs many elements to define and it is hard to elicit elements. For example, if the circumstance is “the classroom is too bright to see the presentation image and it is uncomfortable to study”, then we need an illumination sensor to check the brightness of the classroom, the criteria of “too bright” for the presentation, the temperature and humidity sensor observation values and the criteria for checking the situation being too “uncomfortable to study”.

The UbiSOA [6] and ClickScript [10] require the selection of the relevant sensor, the criteria of the context and the simple operation or code (e.g., AND, OR, greater than, if/then, loops, *etc.*) to define the context. The JIGSAW [11] provides the pre-defined function as jigsaw-puzzle pieces, but it is difficult to define the detailed service features because it does not provide the relational and arithmetic operations. To solve these problems, we provide the pre-defined context such as hot, cold, damp, dry, dark, bright, loud, morning, lunchtime, *etc.* The user can simply select the pre-defined context to define the service condition without considering the required sensor and criteria.

### 5.2. Context Modification and Personalization

People have different criteria according to personal preferences. For example, some people feel cold at a temperature of 2 degrees below zero, but other people do not feel cold at the same temperature. So context modification and personalization is needed. The UbiSOA, ClickScript and JIGSAW do not manage the context per user. So, users should create the new context for context modification at the abovementioned environment. Also, in all related researches—[6,10,11]—the user should change the ontology by the programming language to modify the context. To solve these problems, we create a context as an instance of the *Context* class on the base ontology per user. Therefore, it has different criteria, and we provide the condition-setting phase for the user to modify the context easily through the web-based service modeling UI.

### 5.3. Context Mash-Up

In order to express a variety of service conditions, the context mash-up is needed. The context mash-up provides for reuse and combination of contexts. The UbiSOA provides the context mash-up through the connection between existing contexts, but the ClickScript and JIGSAW create a whole context to create the composited context because it does not support the context mash-up. However, we provide the hierarchical structure with the sub-context to define the composited context. Therefore, the user can create the new composited context through modification of the existing context and the context mash-up.

### 5.4. Reusable Service Element

We need to create a new service through the existing service elements such as service environment, condition and behavior. For example, if a service consists of the service condition *C_1_*, the service behavior *B_1_* in the service environment *E_1_*, and the user wants to add another service behavior *B_2_* in the same service condition *C_1_* and the service environment *E_1_*, the UbiSOA and JIGSAW provide the existing service element as a form of module to reuse. However, the ClickScript should be defined in a single screen without distinction of service elements, so it requires definitions of all service elements, even if some service elements are the same as those in the existing service. Therefore, we provide the reusable service element through the *Place* and *Object* class of the base ontology. We also provide the place- and object-setting phases for the user to modify and reuse the existing service domain through the web-based service modeling UI. It is efficient for changing the service domain.

### 5.5. Sequential Service Modeling

To define the IoT service, the user should consider various service elements such as environment, relevant sensor, criteria of the context, service behavior, related actuator and service procedure. For the general user, it is difficult to consider the priorities of each service element. If the service is more complicated, the user will find it more difficult to consider. Also, the considered service elements and priorities can be different according to the service-processing mechanism of each IoT service platform. Therefore, the user finds it difficult to represent his/her intention and requirement, and the service satisfaction can be lowered. All related researches—[6,10,11]—should define all service elements in a single screen regardless of the service modeling flow. We proposed the user-driven service modeling process with the web-based service modeling UI to provide service elements sequentially. Also, it only shows necessary (or minimized) service elements in each service modeling process to avoid confusion and mistakes of the user. We also arranged the process state bar at the top of screen to recognize the current phase as shown in Figure 12.

### 5.6. Programming Ability Not Required

A non-technical user has no programming ability, such as PHP and JavaScript. Also, they lack the ability to understand the operation or code (e.g., AND, OR, greater than, if, then, for, while, *etc.*). But the UbiSOA demands PHP-based programming work to create the service element module, and it defines the service through the connection between service element modules by PHP language. ClickScript also defines service by making a connection between the service element blocks through the relational operator, and it requires JavaScript programming work to create the new service element block. These methods are difficult for users who are not familiar with the creation of a service flow chart. JIGSAW does not demand programming ability because it represents the service element as a jigsaw-puzzle piece that is a certain image or icon. However, it lacks the diversity of expression about various service elements, and it can be understood differently with the same image or icon according to different people. To solve this problem, we provide the web-based service modeling UI that does not require programming ability. The user can simply select some service elements according to the four service modeling phases: Place setting, Object setting, Object and function selection and Condition setting. Therefore, the user can create the target service through the sequential service modeling.

## 6. Conclusions

This paper has proposed an IoT-based user-driven service modeling environment for a smart space management system. It is an environment aimed at lowering barriers for the non-technical user. Users are able to create their own personalized IoT service without requiring any programming ability. The proposed environment consists of the user, the IoT service market, the IoT service platform and the service spaces. To aggregate context information, we placed RSSG in every service space. The RSSG aggregates, manages and sends the object information to the IoT service platform using an abstracted common format. The IoT service market provided service registration, modification, personalization and brokering to spread the IoT service and the user-participation IoT service ecosystem. Also, it provided the web-based service modeling UI for users to create a personalized IoT service. The IoT service market abstracted the service requirement to deliver to the IoT service platform. The IoT service platform applied this requirement to the proposed base ontology for context awareness. It processed the context information and service requirement to provide the IoT service through the ontology-based context-information processing. The IoT service platform controls the actuators according to the service requirement. Also, we presented the comparison results with UbiSoA, ClickScript and JIGSAW. According to the comparison results with the analyzed six features (predefined context, context modification and personalization, context mash-up, reusable service element, sequential service modeling and programming ability not required), we showed that the IoT-based user-driven service modeling environment satisfies all features.

As a result, through the proposed environment, users can create their own IoT service without any programming ability. Also, the user is able to modify and personalize the IoT service through the user-centric and participatory paradigm. Our future work includes the richer expression of the service modeling environment for expert users and a way of reconstructing the existing IoT service. Also, service quality and performance evaluation have not been discussed in this paper. We will consider those issues to enhance and ensure our work in the future.

## Figures and Tables

**Table 1.** Object-data format.

**Sensor**		**Actuator**	
	
**URI**	/sensor/tempsensor1	**URI**	/actuator/lamp2
**Type**	Temperature	**Name**	Lamp
**Unit**	centigrade	**Status**	ON
**Value**	28	**Function list**	Turn on, Turn off
**Accuracy**	5	**Location**	-
**Location**	-	**Ownership**	Jun
**Ownership**	Jun & Jun's Family		

**Table 2.** Comparison features analysis.

**No**.	**Comparison Features**	**UbiSOA** [6]	**ClickScript** [10]	**JIGSAW** [11]	**Proposed Approach**
1	Predefined context	No	No	Yes	Yes
2	Context modification and personalization	No	No	No	Yes
3	Context mash-up	Yes	No	No	Yes
4	Reusable service element	Yes	No	Yes	Yes
5	Sequential service modeling	No	No	No	Yes
6	Programming ability not required	No	No	Yes	Yes
